# Fluid overload–associated large B-cell lymphoma with primary biliary cirrhosis: A case report

**DOI:** 10.3389/fonc.2023.1145540

**Published:** 2023-03-31

**Authors:** Huan Wang, Quan Zhang, Qin Liu, Xian Wu, Ke Ma

**Affiliations:** Department of Infectious Diseases, The Affiliated Hospital, Guizhou Medical University, Guiyang, China

**Keywords:** lymphoma, fluid overload–associated large B-cell lymphoma, primary biliary cirrhosis, PET/CT, ascites, bone marrow biopsy, case

## Abstract

The 5th edition of World Health Organization Classification of Haematolymphoid Tumours (WHO-HAEM5) is characterized by its hierarchical system for classification and novel entities/types. Considering the significant discrepancy in clinical manifestations and prognosis, fluid overload–associated large B-cell lymphoma (FOALBCL) has been a new addition to the WHO-HAEM5, being distinct from the traditional diagnosis of primary effusion lymphoma. In this manuscript, we report a patient who was diagnosed with FOALBCL that a novel entity introduced in the WHO-HAEM5. It is an instance of a successful application of the updated WHO-HAEM5 and indicates that the ′Blue Book′ could confer convenience and benefits on both patients and clinicians. Moreover, the patient combined primary biliary cirrhosis (PBC), which is a relatively rare condition compared to the underlying medical condition of viral cirrhosis. Due to atypical clinical symptoms and invasive biopsy of lymphoma, sometimes, diagnoses might be undesired, which eventually leads to a poor prognosis. With this case report, it reminds not just hematologists but also other specialists to pay attention to the updates and revisions of the classifications of lymphoma.

## Introduction

Fluid overload–associated large B-cell lymphoma (FOALBCL), a novel entity/type, was first introduced in the 5th edition of the World Health Organization Classification of Haematolymphoid Tumours (WHO-HAEM5) published in 2022 (1). The updated WHO ‵Blue Book′ identified the new addition entity from ambiguous and confusing notions, such as “primary effusion lymphoma (PEL)–like lymphoma” or “KSHV/HHV8-unrelated PEL-like lymphoma” (2, 3). Compared to PEL, which is characterized by large pleomorphic cells with differentiation grades from immunoblastic to anaplastic, FOALBCL demonstrates similar cytomorphologic detection and immunophenotype (CD20 and CD19) but is quite distinct in viral features (negative of KSHV/HHV8), demographics (elderly individuals with underlying conditions causing fluid overload), and clinical outcome (less aggressive) (2). Moreover, PEL listed as the unaltered entity was categorized as KSHV/HHV8-associated B-cell lymphoid proliferations and lymphomas, concomitant with other entities such as KSHV/HHV8-associated multicentric Castleman disease (KSHV/HHV8-MCD), germinotropic lymphoproliferative disorder (KSHV/HHV8-GLPD), and KSHV/HHV8-positive diffuse large B-cell lymphoma (KSHV/HHV8-DLBCL), while FOALBCL belongs to a large B-cell lymphoma family in the WHO-HAEM5 (1). Taken together, a quantity of entities have been generally arranged in the WHO-HAEM5 with accuracy and accessibility to prevent the overdiagnosis of lymphoma, owing to the rapid development in the understanding of lymphoid proliferations.

Here, we report a case of a man with the admission diagnosis of decompensated cirrhosis. In the period of hospitalization, the diagnosis did not account well for the patient’s clinical presentation, laboratory results, and therapeutic efficacy. Due to the critical evidence of the ascites cytological test and immunohistochemical examination, we modified the initial diagnosis to FOALBCL, which was first introduced in the WHO-HAEM5. The case report is aim to enhance understanding on the diagnosis of FOALBCL, particularly in the presence of confounding for similar clinical and laboratory characteristics.

## Case description

We report the case of a 70-year-old man who presented recurrent abdominal pain and distention, accompanying skin irritation and pruritus for more than 9 months. The patient denied a family history or special occupational exposures, without drinking history, and was admitted to hospital with a possible diagnosis of decompensated cirrhosis.

**Figure 1:**
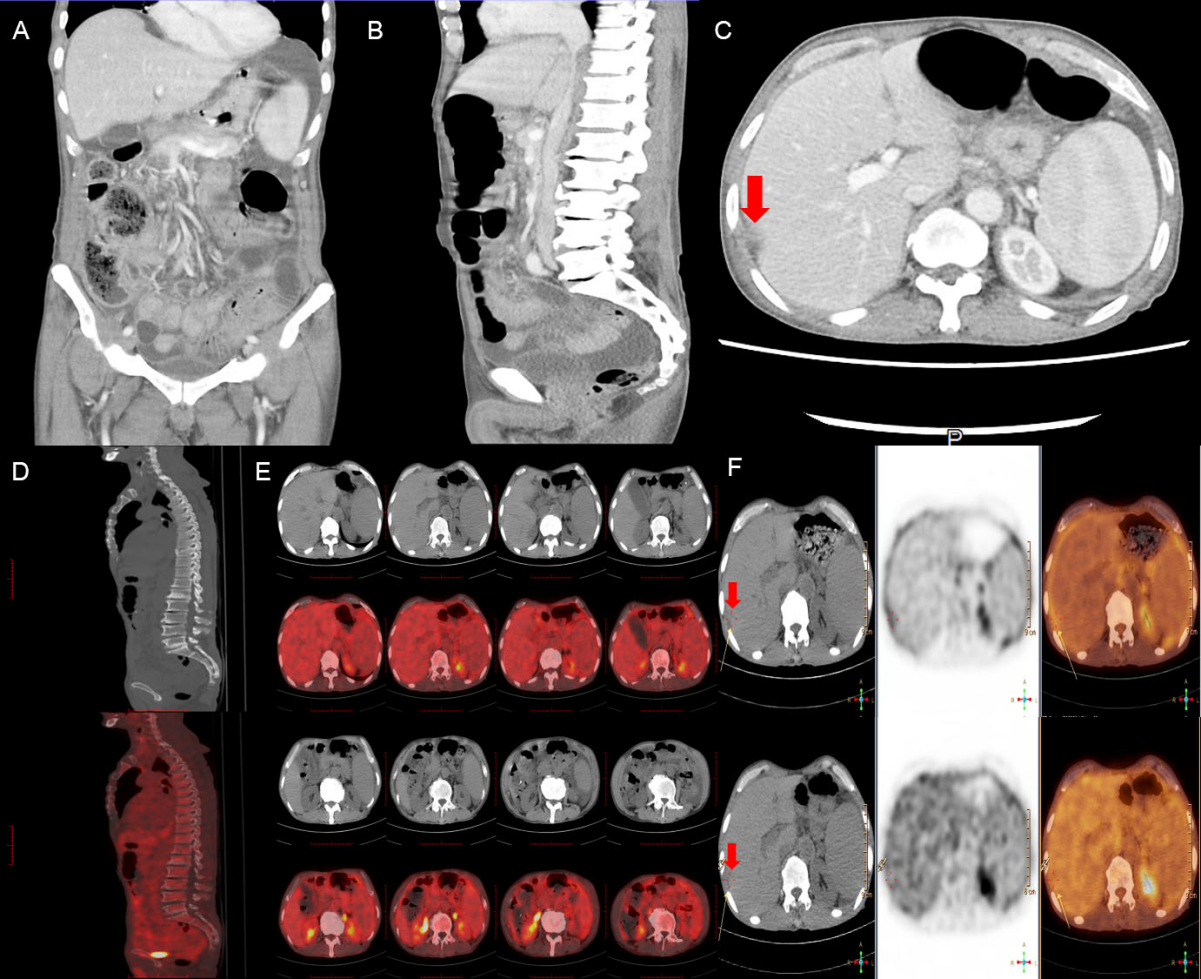
Contrast-enhanced abdominal CT **(A-C)** scan showed volumetric disproportion of lobar and segmental of the slightly enlarged liver with massive abdominal-pelvic fluid accumulation. Full-body PET-CT scan **(D-F)** showed no significant hypermetabolism but a slightly low signal from the subcapsular area in the right hepatic lobe with ascites and splenomegaly. Red arrows point to the subcapsular area in the right hepatic lobe with ascites.

On admission, physical examination revealed abdominal bulge and tenderness with shifting dullness but without jaundice and caput medusa. An abdominal ultrasound revealed multicavity effusion, especially massive peritoneal effusion. Chest-computed tomography (CT) scan showed a limited amount of pleural effusion, and contrast-enhanced abdominal CT indicated early-stage cirrhosis and splenomegaly complicated by abdominal-pelvic fluid accumulation. Beyond these, a subcapsular effusion of the right liver lobe, gastric wall edema lesion, and subcutaneous edema were in the imaging (shown in **Figure 1**). Further full-body PET-CT images correspond to contrast-enhanced abdominal CT and disclosed no significant hypermetabolism (shown in **Figure 1**). Blood tests excluded multiple organ failure and potential virus infection, which was caused by hepatitis B, C virus (HBV, HCV), Epstein–Barr (EB) virus, and Kaposi sarcoma herpesvirus (KSHV)/human herpesvirus-8 (HHV8) virus. The investigation of liver function showed normal hepatic enzymes in alanine aminotransferase (ALT) 13.90 U/L, aspartate aminotransferase (AST) 32.40 U/L, total bilirubin (TBIL) 9.06 umol/L, direct bilirubin (DBIL) 3.43 umol/L, indirect bilirubin (IBIL) 5.63 umol/L, and slightly increased alkaline phosphatase (ALP) 101.0 IU/L and gamma-glutamyltranspeptidase (GGT) 68.9 IU/L, while there was an obvious reduction of total protein (TP) 51.72 g/L and albumin (ALB) 24.21 g/L. A representative antibody for primary biliary cirrhosis (PBC), antimitochondrial M2 (AMA-M2), was positive. Routine blood tests indicated anemia and thrombocytopenia with red blood cell count (RBC) 3.07 × 10^12^/L, hemoglobin (Hb) 88 g/L, and platelet count (PLT) 115.0×10^9^/L. Furthermore, a slight elevation in high-sensitivity cardiac troponin T (hs-cTnT) 0.019 ng/ml was also noted.

**Figure 2:**
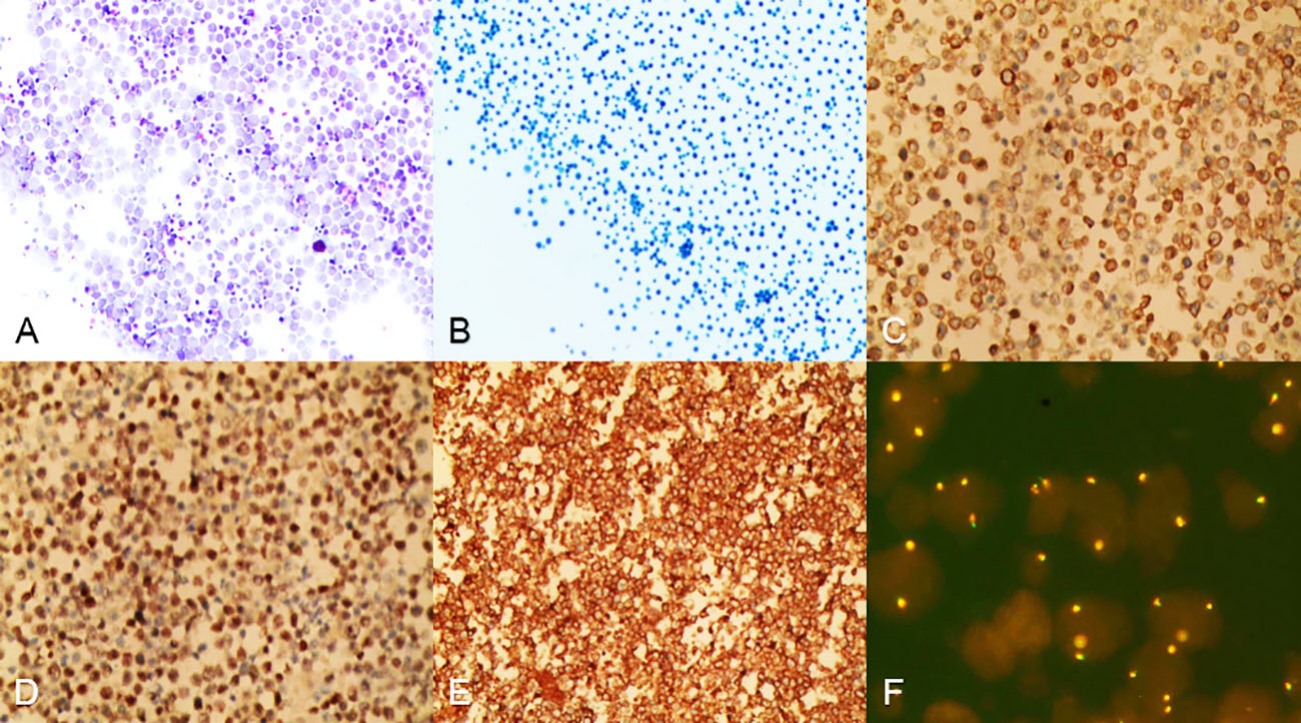
Cells from ascites were centrifuged and then stained. **(A, B)** Heterogeneous lymphoid-like cells were distributed in a scattered pattern, hematoxylin–eosin staining in A, papanicolaou staining in B, magnification ×100. **(C-E)** Ascites cells were positive for CD20, CD79α, LCA, magnification ×100. **(F)** FISH analysis revealed that 20%–30% of the ascites cells exhibited c-MYC amplification, magnification ×100.

**Figure 3:**
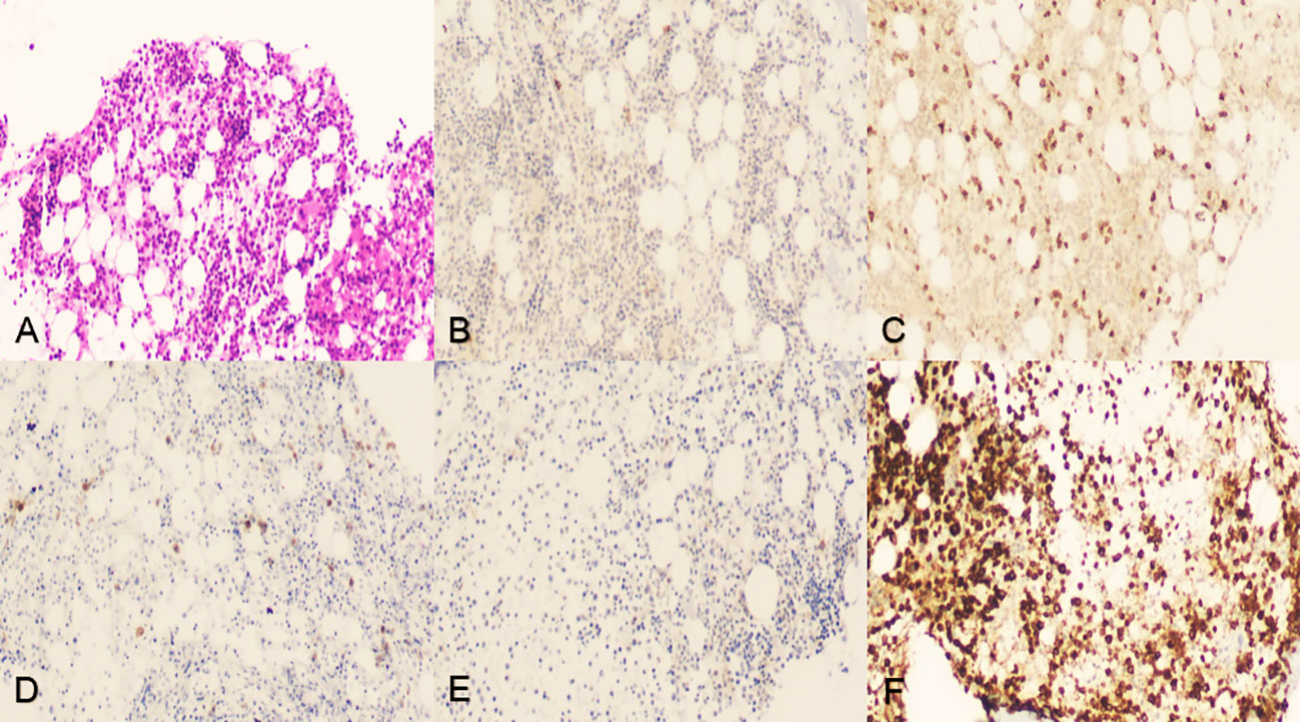
Iliac crest bone marrow aspiration and biopsy. **(A)** Tissue section indicated neither lymphocytic infiltration nor myelodysplastic disorder but showed abnormal immunohistochemistry, hematoxylin–eosin staining, original magnification ×100. **(B-D)** Tumor cells were positive for CD20, CD3, and CD79α, original magnification ×100. **(E)** Tumor cells were negative for BCL-6, original magnification ×100. **(F)** Tumor cells were positive for MPO activity, 3,3’-diaminobenzidine staining, original magnification ×100.

In an attempt to a definitive diagnosis, ascites and marrow were examined. The biochemical test of ascites found an abnormally high in lactate dehydrogenase (LDH) 4538.0 IU/L. Histology suggested that the ascites puncture cytology contained aggressive of large-sized B lymphocytes positive for CD20, CD79α, LCA, c-MYC (shown in **Figure 2**), and other biomarkers, such as PAX5(+), CD3(+), CD19(+), CD34(-), HHV8(-), Ki-67(+, 60%), BCL-6(+), MUM1(-), sox11(-), EMA(-), and MPO(+) consistent with the result of the immunohistochemical staining of bone marrow biopsy, which presented CD20(+), CD79α(+), CD3(+), BCL-6(-), MPO(+) (shown in **Figure 3**), CD10(-), Ki-67(+, 1%<), and MUM(-).

According to available information, a cogitative diagnoses, FOALBCL, of this patient was produced. All symptoms could be completely recovered and vanished after peritoneal drainage by percutaneous puncture catheterization. Liver dysfunction returned to normal (ALT 10.40 U/L, AST 21.40 U/L, TBIL 15.16 umol/L, DBIL 5.66 umol/L, IBIL 9.50 umol/L, ALP 79 IU/L, GGT 28.13 IU/L) and hypoproteinemia was improved (TP 64.30 g/L, ALB 35.20 g/L) after receiving albumin prepared from human plasma injection (total 130 g). Despite the fact that anemia has not been corrected (RBC 2.53 × 10^12^/L, Hb 77 g/L, PLT 103.0 × 10^9^/L), myocardial enzyme indicators have normalized (hs-cTnT 0.013 ng/ml) without any targeted-oriented therapy. Over 1-monthly symptomatic and supportive treatment, the patient made a discharge decision for seeking subsequent therapy by a local hospital. Before compiling the data and writing up this manuscript, informed consent and subsequent therapies have been confirmed by conducting a telephone follow-up on the patient. Follow-up information suggested that the patient was still alive and was undergoing chemotherapy smoothly without apparent adverse effects.

## Discussion

The said data strongly suggest that the modified WHO-HAEM5, which facilitates a pragmatic approach to distinguish and diagnose the less aggressive FOALBCL with a fairly favorable prognosis from PEL, may assist in the diagnosis and treatment process of the patients with similar clinical symptoms. In conventional wisdom, detecting the evidence of viral infection by KSHV/HHV8 in the neoplastic cells is essential for the diagnosis of PEL, which meanwhile provides a reliable basis for distinguishing FOALBCL (4). It is the state that a combination of immunodeficiency and KSHV/HHV8 has contributed to the aberrant pattern of gene rearrangements and phenotype heralding a poor prognosis (5). A meta-analysis demonstrated that a median of 8 months and >1-year survival rate 42.1% in KSHV/HHV8-negative PEL (now classified as FOALBCL) versus 4 months and 17.3% in PEL, respectively (6). Noteworthy, it is a more reliable diagnosis of FOALBCL that exclusion at the stage of KSHV/HHV8 infection combines with aberrations in B-cell-specific markers CD20 (6, 7) and PAX5 (8). Relevant key points were seen in this case report, even though no lymphocytic infiltration or myelodysplastic disorder were detected in bone marrow biopsy. Similar to other studies report in literature (9, 10), our patient obtained complete remission with effusion drainage alone. In addition, a quantity of cases reported previously verified that underlying lymphoma is not the leading cause of death in FOALBCL (2). Thus, effectively, symptomatic managements for fluid overload state play a pivotal role, such as vasoactive drugs and diuretic for heart failure and pleurodesis for pleural effusion (11–14). However, it is because of the availability of symptomatic strategy that clinicians will be likely to overlook the diagnosis of FOALBCL, which exerts a detrimental effect on the long-term outcome.

The underlying medical condition of cirrhosis is another issue worthwhile exploring. The previous study showed that the medical conditions of cirrhosis caused by hepatitis C (15, 16) and heart failure have been involved in the pathogenesis of fluid overload states and early onset of symptoms (17). Chak et al. found that KSHV/HHV8-negative effusion-based lymphomas (now referred to as FOALBCL) existed a magnitude higher rate of 26.5% coinfection with HCV than the baseline prevalence rate of 2.0% in the general population of the United States (18). Obviously, these features were not present in our case. In light of dermatological signs, abdominal CT imagine, positive serology of AMA-M2, and raised ALP/GGT, we preferred PBC leading to early-stage cirrhosis that has promoted the development of fluid overload states. We have been inspired by the present case and draw some insight that cirrhosis caused by autoimmune or viral hepatitis might confound and fragment the initial diagnosis of FOALBCL due to unidentified ascites, which should be taken seriously by more hematologists.

Overall, FOALBCL as a new entity introduced in the WHO-HAEM5 brings a lot of diagnostic convenience for this patient, yet several points are worthy of discussion. We need to specify that the WHO-HAEM5 was released before a consensus drawn by the clinical advisory committee (CAC); meanwhile, a more prudent guideline has been formulated and published by the International Consensus Classification (ICC). According to the ICC, HHV8 and EBV-negative primary effusion-based lymphoma were identified as a provisional entity, which needs more reliable and clear diagnostic criteria to establish under considerations by the CAC, is equivalent to FOALBCL entity in the WHO-HAEM5. Taking a salient example, excluding EB virus infection, which displays a high correlation with diffuse large B-cell lymphoma, is a required criterion for the diagnosis of HHV8 and EBV-negative primary effusion-based lymphoma, whereas it is not for FOALBCL. Further explorations regarding the classifications of lymphoma deserve more attention and expectation for superior clinical utility.

## Data availability statement

The original contributions presented in the study are included in the article/supplementary material. Further inquiries can be directed to the corresponding author. 

## Ethics statement

The studies involving human participants were reviewed and approved by Ethics Committee of Affiliated Hospital of Guizhou Medical University. The patients/participants provided their written informed consent to participate in this study. Written informed consent was obtained for the publication of this case report.

## Author contributions

HW and KM conceived the study. HW, QZ, QL, and XW generated and interpreted data. KM wrote the manuscript. All authors contributed to the article and approved the submitted version. 
